# Symptomatic Hepatoduodenal Adenomas Treated With Conventional Radiation Therapy in a Patient With Familial Adenomatous Polyposis: A Case Report

**DOI:** 10.1016/j.adro.2023.101181

**Published:** 2023-01-16

**Authors:** Hayden Ansinelli, Shona T. Dougherty, Cynthia Rodriguez, Uma D. Goyal

**Affiliations:** a Department of Radiation Oncology, University of Arizona, Tucson, Arizona; b Banner MD Anderson Cancer Center, Phoenix, Arizona

## Introduction

Familial adenomatous polyposis is an inherited autosomal-dominant syndrome resulting from germline mutations in the *APC* gene, which predisposes patients to an extremely high risk of developing colorectal cancer throughout their lives.^1^ Although prophylactic colectomy reduces the lifetime risk of colorectal cancer development, patients remain at elevated risk of extracolonic manifestations, such as formation of adenomas throughout the upper gastrointestinal tract.^1^^,^^2^ Although these lesions may be benign or malignant, patients can develop significant adverse sequelae from growing adenomas via mass effect on nearby structures and often require procedural intervention.^3^^,^^4^ Radiation therapy (RT) has been used in the definitive and palliative settings for a variety of gastrointestinal and hepatobiliary malignancies; however, its potential utility for benign duodenal adenomas remains unclear.^5^^,^^6^ Here, we describe our results of using conventionally fractionated RT for 2 symptomatic hepatoduodenal adenomas causing severe abdominal pain.

## Case Introduction

Patient was a 77-year-old female with a history of familial adenomatous polyposis treated with total colectomy and end-ileostomy at age 18, as well as multiple laparotomies for gastric/duodenal polyp removals over the following several decades. She continued close follow-up with the gastroenterology department over her lifetime, endorsing a persistent multiple-year history of dull, achy epigastric pain, which was attributed to her numerous previous surgeries.

She began to experience new, progressively worsening, sharp “stabbing-like” pains in her right upper quadrant. The sharp pains had become so severe that she would feel her “body locking up,” and it would interrupt her from being able to speak, walk, maintain focus on any topic, and even limited her ability to breathe normally during the duration of the attack. The episodes initially occurred only 1 to 2 times per day, but at time of presentation, she reported a frequency increase to more than 20 attacks per day and a significant effect on her quality of life. The attacks were unrelated to dietary intake and were only alleviated by time, lasting anywhere between 5 and 30 seconds. She presented to the hospital in late 2019 for a particularly bad series of painful attacks, where an abdominopelvic contrast computed tomography scan showed intrahepatic biliary duct dilatation and an abnormal increased density noted within the common hepatic duct and extending into the proximal common bile duct.

**Figure 1:**
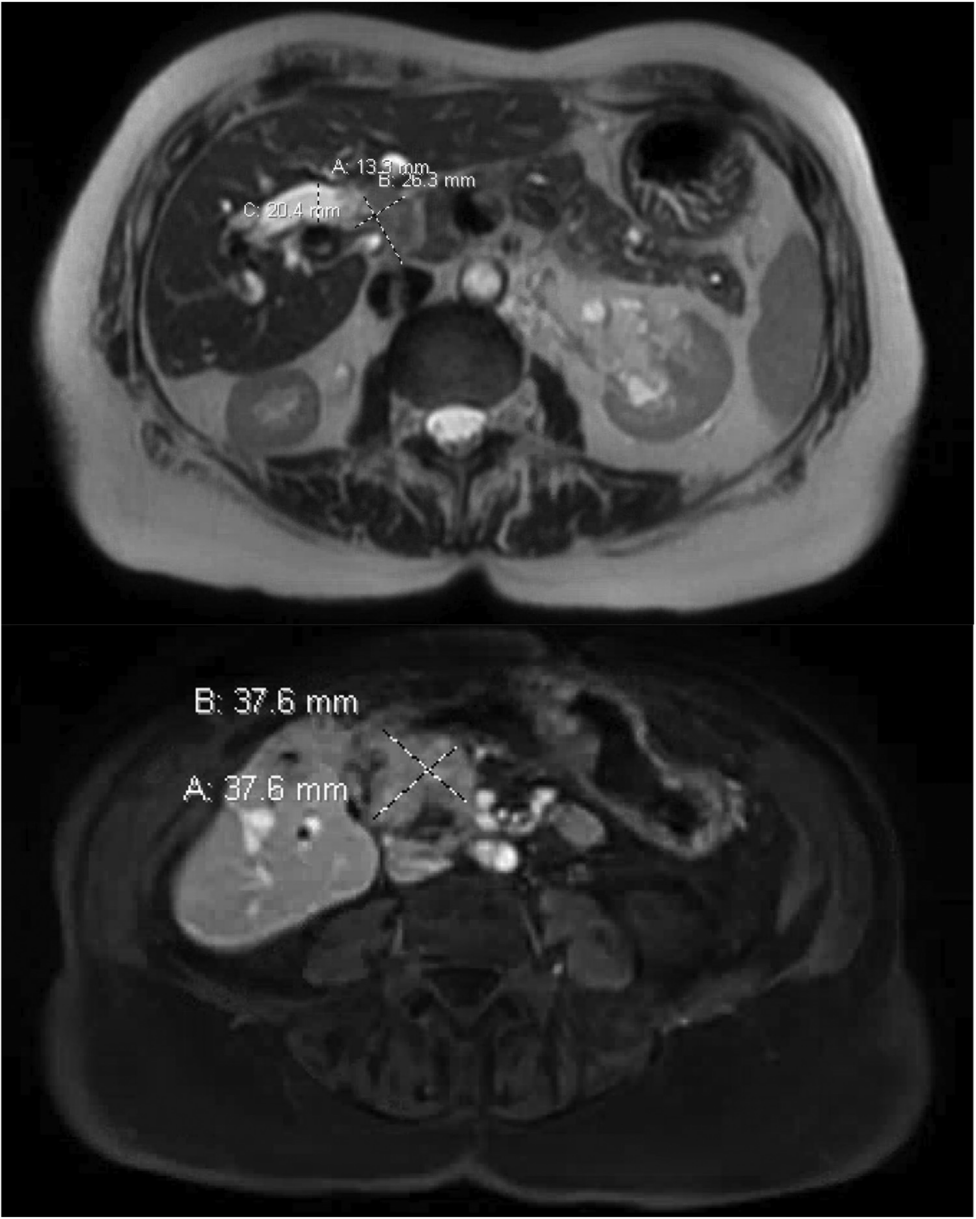
Pretreatment magnetic resonance imaging (2 months before radiation therapy start date) demonstrating the ill-defined exophytic lesion measuring 3.8 × 3.8 cm in the papilla.

**Figure 2:**
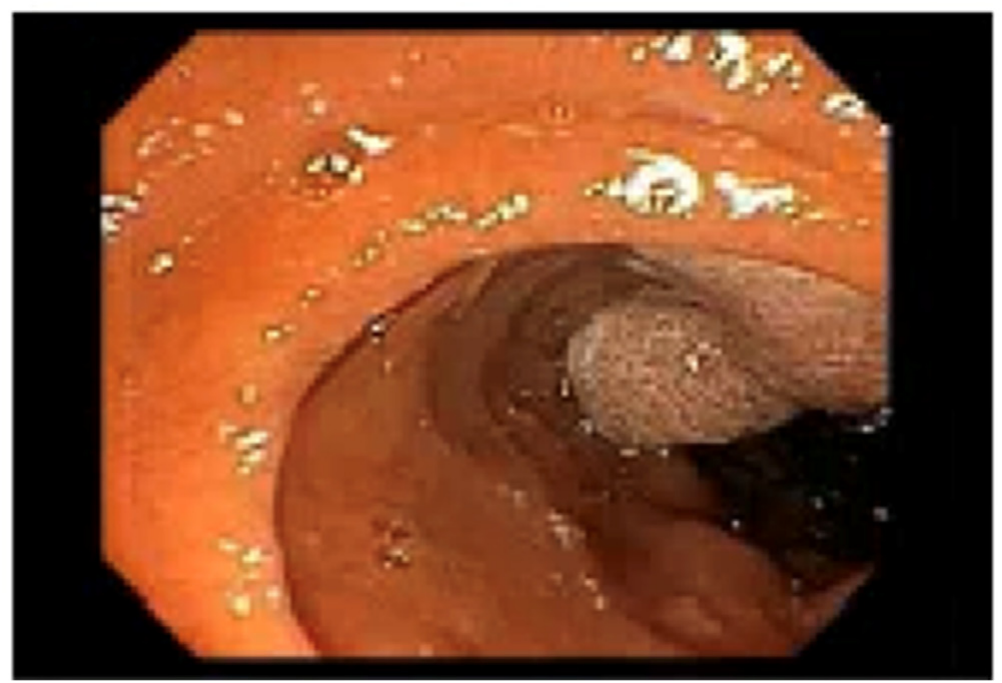
Pretreatment esophagogastroduodenoscopy and endoscopic retrograde cholangiopancreatography demonstrating a villous, nonbleeding, and sessile mass consistent with an adenoma in the major papilla.

**Figure 3:**
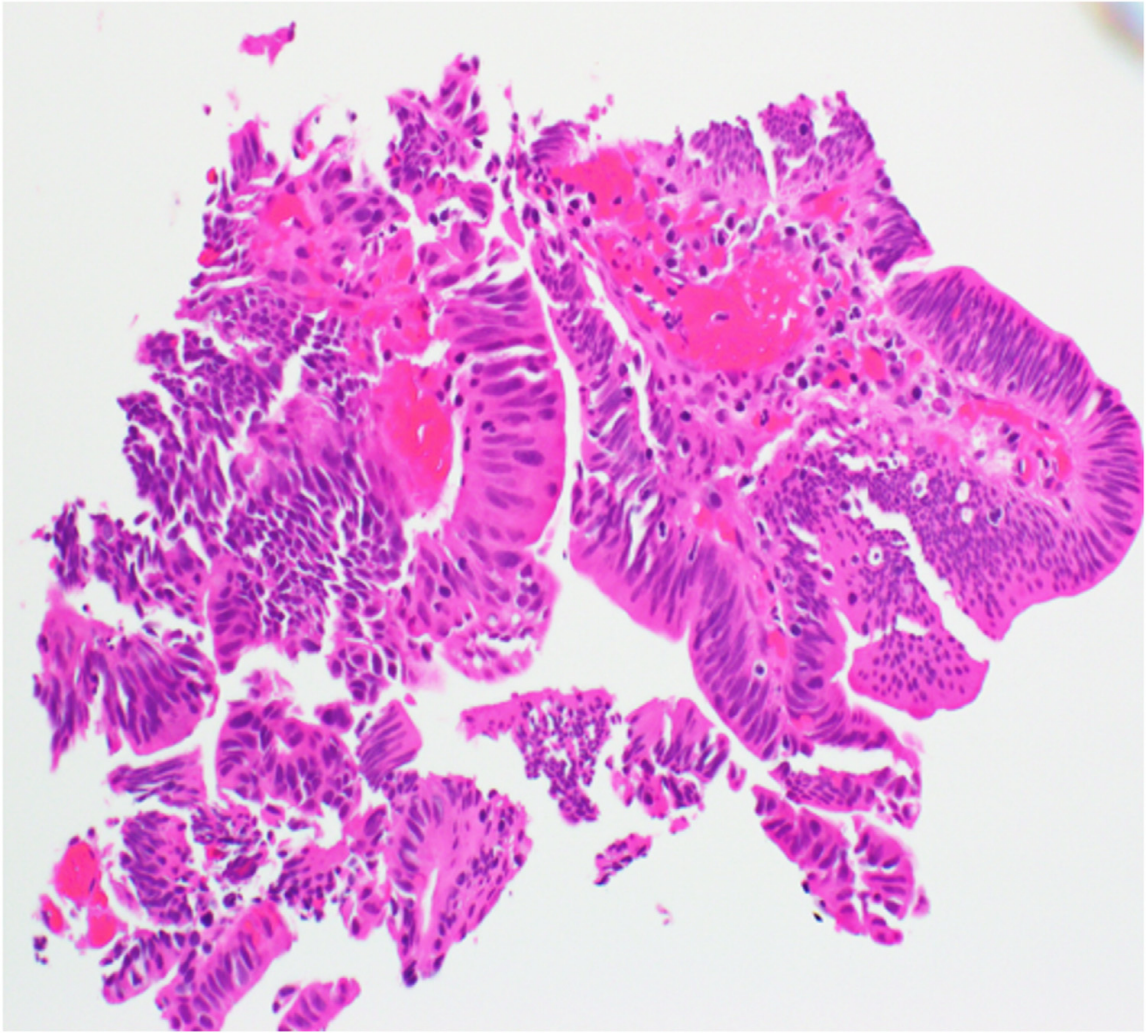
Pathology slide from biopsy of the bile duct mass, consistent with adenomatous polypoid epithelium and superficial fragments of biliary mucosa with extensive acute inflammation and adenomatous changes, and no identifiable high-grade dysplasia or malignancy.

She was discharged on hydrocodone and scheduled for outpatient esophagogastroduodenoscopy (EGD), which subsequently revealed a 3-cm polypoid mass in the duodenum. This was followed by magnetic resonance imaging (MRI) of the abdomen, which redemonstrated the mass in the duodenum measured at 3.8 × 3.8 cm, and another 2.6- × 2.0-cm mass at the liver hilum causing compression of the common bile duct (Fig. 1). She subsequently underwent additional EGD and endoscopic retrograde cholangiopancreatography (ERCP), during which a polypectomy was performed and a pancreatic stent was placed (Fig. 2), with pathology revealing a tubulovillous adenoma (Fig. 3). No high-grade dysplasia nor malignancy was identified.

Her MRI was reviewed at the institutional tumor board, and there were concerns for malignancy, given different signal characteristics in the mass compressing the common bile duct compared with the ampullary lesion. She thus underwent additional ERCP and biopsy in April 2020, with pathology revealing adenomatous polypoid epithelium and no evidence of high-grade dysplasia nor malignancy. Despite evidence of only a tubulovillous adenoma being demonstrated on her pathology, there was concern for sampling error and the possibility of underlying malignancy. Given her numerous previous surgeries, the multidisciplinary consensus was that additional surgical resection would be difficult because of the extent of anticipated adhesions, and reconstruction would be challenging or impossible, given potential intrahepatic extension of the lesion.

She was then referred to the interventional gastroenterology department for consideration of ablation. It was felt that the lesions were too large for effective endoscopic ablation. The patient voiced a preference for nonoperative treatments, and she next presented to the radiation oncology department for consideration of RT options. Given the scarcity of literature on RT for duodenal adenomas, dose selection was guided predominantly by the dose the response of benign adenomas in other sites (such as the pituitary gland), which tend to respond well to conventionally fractionated RT doses in the 50- to 54-Gy range.^7^

## Treatment Plan

**Figure 4:**
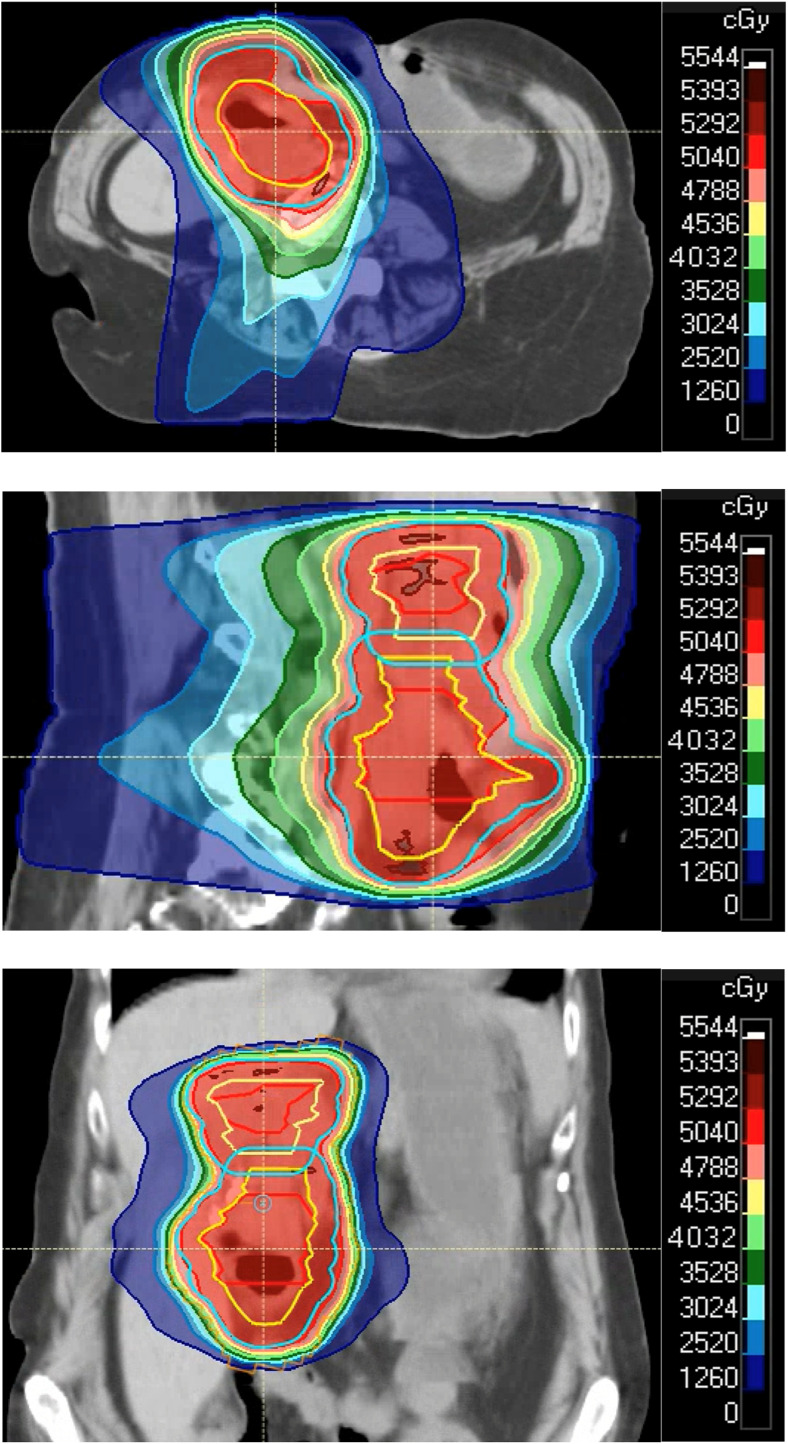
Visual depiction of the used treatment plan (intensity modulated radiation therapy) for the 2 lesions, shown in axial, sagittal, and coronal planes with dose distributions.

A treatment plan of 50.4 Gy in 28 fractions was agreed on for the duodenal and perihepatic lesions, with the use of intensity modulated RT for sparing of nearby critical structures (Fig. 4). Alternative shorter fractionation regimens were considered, but the patient voiced preference for the conventional treatment course, given the combination of her overall moderate performance status, worsening pain level, and lack of other nonprocedural treatment options if unsuccessful.

The patient was instructed to take prophylactic ondansetron a half hour before simulation and avoid food intake for 2 hours before her scan and subsequent treatments. She underwent computed tomography simulation in the supine position, without intravenous contrast due to difficulty obtaining access on the day of simulation. Gross tumor volumes for the liver hilum and duodenum were contoured with assistance from her most recent MRI, which was fused to her simulation scan. An internal target volume was created to account for lesion movement with her respiratory cycle, and a 1-cm expansion was added to the internal target volume for her planning target volume.

## Treatment Course

Overall, the patient tolerated treatment as expected for a patient receiving fractionated RT to the abdomen. Her only attributable symptoms were intermittent fatigue, relieved by rest, and mild nausea, for which she used daily oral ondansetron on treatment days for prophylaxis and symptom reduction. Her pain response was tracked closely, daily by the patient herself, and weekly during on-treatment visits with the attending physicians and nursing staff.

Notably, on her second day of treatment, she experienced 2 of her attacks during an approximately 30-minute discussion, which had not been witnessed previously by clinical staff. The attacks appeared to last about 20 seconds each, during which they were so severe that she was forced to stop conversation and her body remained in a locked, stiff position. Although she was shaken, she reported her pain subsided afterward, and then returned to her baseline “dull, achy pain” level that she had endorsed for years and attributed to her numerous previous surgeries.

After her seventh treatment, she reported that her attack severity was unchanged, but she felt there was a decrease in overall frequency. On treatment day 11, she reported that she noticed that the pain character had changed, with slight improvement and more-noticeable radiation of the pain into the midback as opposed to strict localization in the right upper quadrant. On treatment day 19, she reported that her attack frequency and severity both were noticeably lesser than her pretreatment baseline, with further improvement noted on her final day of RT.

She was first seen for posttreatment follow-up at 38 days after completion of RT. She endorsed persistent fatigue, which she attributed to her radiation treatment, but reported she was no longer experiencing any acute episodes of the sharp right upper quadrant abdominal pain. She continued to endorse the constant, dull, crampy epigastric pain that she had endured for years. She reported intermittent, progressively improving nausea well-controlled with ondansetron and no changes to her diet tolerance or bowel habits from baseline.

**Figure 5:**
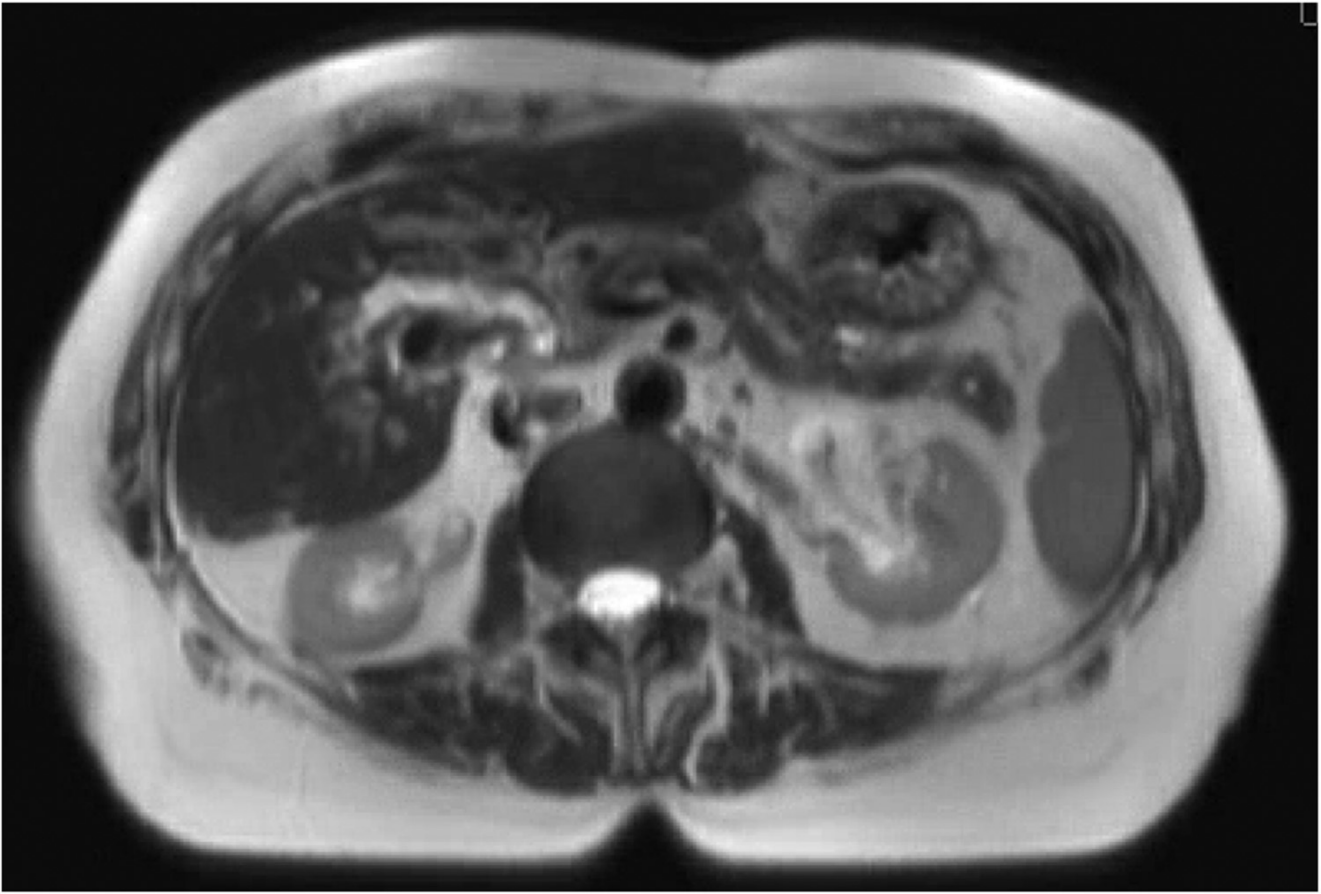
Three-month posttreatment axial T2 magnetic resonance imaging demonstrating interval resolution of the hepatic and duodenal masses, with appreciable edema in the portal region.

At 90 days posttreatment, she underwent MRI of the abdomen and pelvis, which demonstrated postradiation changes along the porta hepatis and surrounding the duodenum with extensive edema (Fig. 5). Neither the previously demonstrated duodenal adenoma at the ampulla nor the perihepatic mass could be clearly visualized, but persistent biliary tract dilatation was noted. During the first 90 days after treatment completion, she experienced 1 total episode of the acute, sharp abdominal pain and reported that it was not as intense as her episodes before RT.

**Figure 6:**
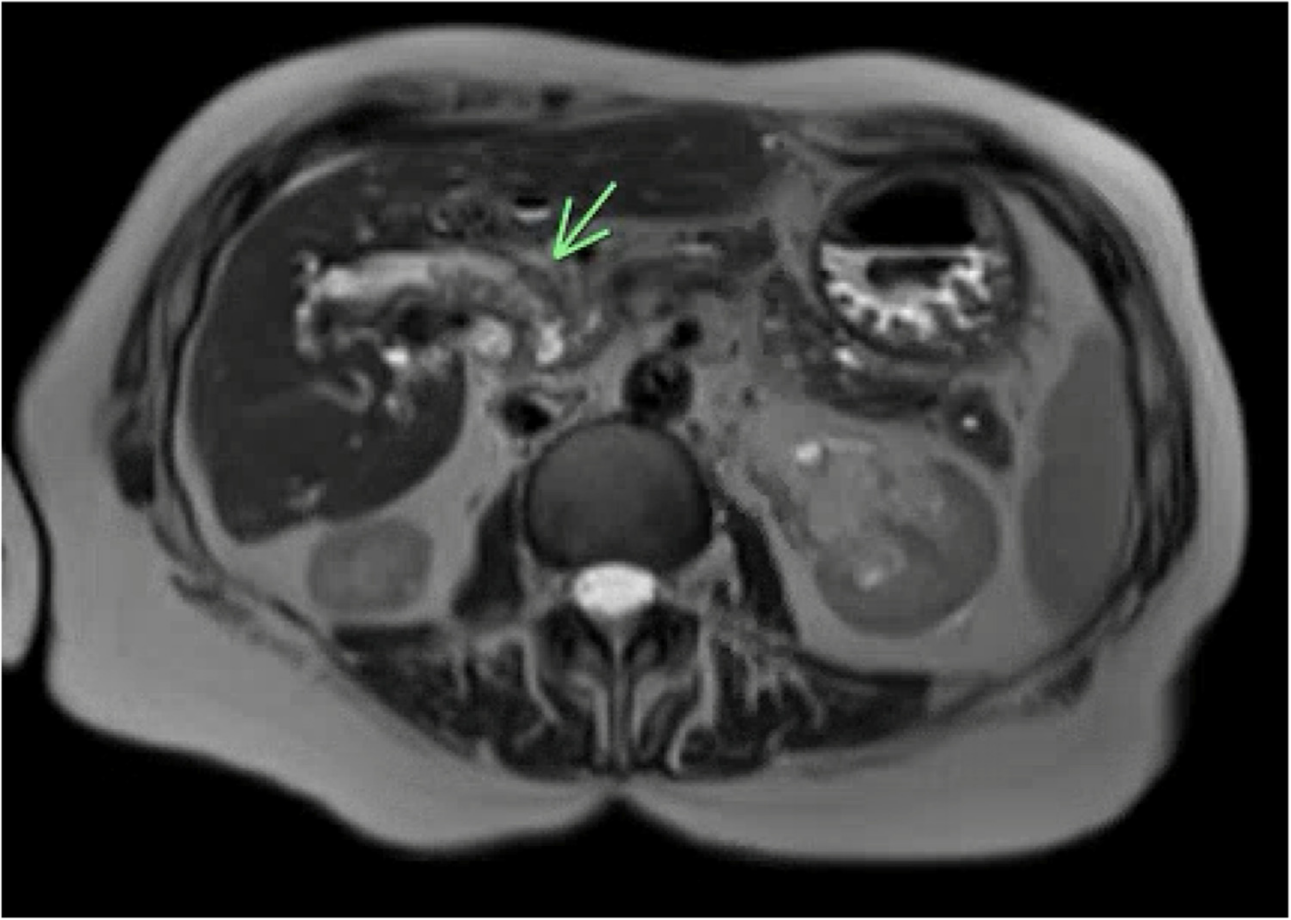
Posttreatment magnetic resonance imaging 6.5 months after treatment (axial T2 sequence) demonstrating interval resolution of the hepatic mass, but findings were concerning for ascending cholangitis.

A second MRI was performed at 6.5 months after completion of RT, which showed marked edema in the hepatic hilum and proximal duodenum likely related to postradiation changes (Fig. 6). Features concerning for ascending cholangitis also were noted, with biliary wall thickening and enhancement, and substantial debris in the common hepatic duct.

Shortly after her second MRI, she presented to the emergency department with epigastric pain and was admitted for evaluation of a suspected upper-gastrointestinal bleed and cholangitis. She underwent EGD + ERCP, which revealed filling defects on the cholangiogram, and she underwent metal stent placement in her common bile duct, and plastic biliary stent placement in the left hepatic duct. Telangiectatic changes were noted in the duodenal–jejunal region as well, suggestive of postradiation treatment effect (image available in Fig. E1).

She followed up with the radiation oncology clinic the next week, at approximately 7 months after treatment completion. She reported feeling much better, with significant improvement in her pain level (including the long-term, dull pain she had previously described) after the stent placement.

Unfortunately, her follow-up after 8 months among all multidisciplinary oncology teams was limited, as she experienced a cerebrovascular accident believed to be caused by poor adherence to an anticoagulation regimen around the time of a heart valve placement. She was left with persistent right-sided weakness and became wheelchair-bound, and died nearly 18 months after completion of her radiation treatment.

## Conclusion

To the best of our knowledge, this is the first report to date of the use of definitive intensity modulated RT to treat symptomatic hepatoduodenal adenomas in a patient with familial adenomatous polyposis who was deemed a poor candidate for surgical or ablative measures. In summary, the treatment was very effective at ameliorating her pain, with posttreatment imaging demonstrating resolution of her lesions.

Although RT was well-tolerated in the acute setting, treatment may have contributed to the development or worsening of symptomatic cholangitis, necessitating stent placement at approximately 6.5 months after RT. The exact contribution of RT to her cholangitis is difficult to describe, given the notable bile duct dilatation noted before initiation of RT; however, post-RT cholangitis has been documented in previous cases of patients receiving upper abdominal radiation.^8^^,^^9^ Given the extent of her treatment fields, which encompassed a substantial portion of the hepatoduodenal region, it is reasonable to assume that radiation may have aggravated her chronic cholangitis to the point of needing procedural intervention.

Overall, the patient was very satisfied with the outcome of the treatment and appeared to be doing well after the stent placement, but the benefit was unfortunately limited by a cerebrovascular accident at approximately 8 months after RT that led to a significant decline in her performance status. Our case study supports the utility of conventionally fractionated RT for the treatment of symptomatic hepatoduodenal adenomas. Although treatment can be delivered at conventional fractionation to 5040 centigray in 28 fractions and was well-tolerated and effective, we encourage consideration of deescalated and shorter-course palliative regimens, as the patient demonstrated a significant clinical treatment response by her third week of radiation treatment.

## Appendix Supplementary materials

Image, application 1

## Footnotes

Supplementary material associated with this article can be found in the online version at doi:10.1016/j.adro.2023.101181.
