# Supplementary material for: Symptomatic Hepatoduodenal Adenomas Treated With Conventional Radiation Therapy in a Patient With Familial Adenomatous Polyposis: A Case Report
Source: Adv Radiat Oncol. 2023 Jan 16;8(3):101181. doi: 10.1016/j.adro.2023.101181 (PMC9971027; doi:10.1016/j.adro.2023.101181)

Supplemental Figure 1. Post-treatment esophagogastroduodenoscopy (EGD) demonstrating resolution of the mass and telangiectatic changes suggestive of post-radiation changes.


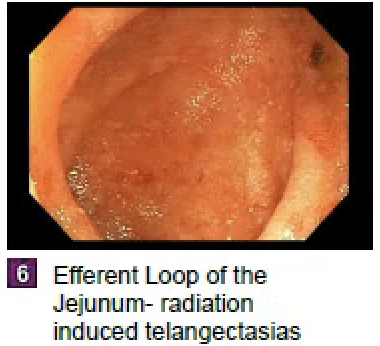


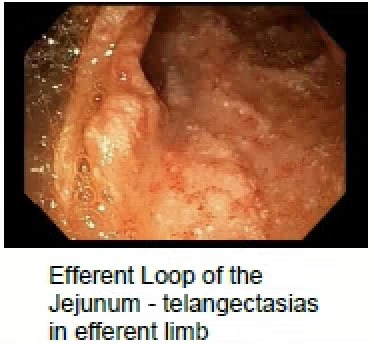


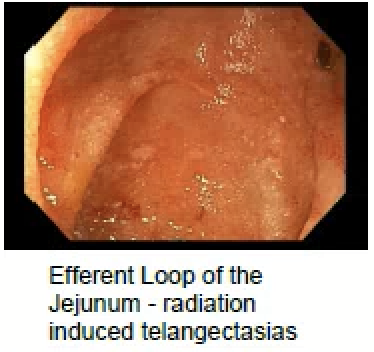

Supplement: Supplementary file 1 [file mmc1.docx]
